# Mortality and Advanced Support Requirement for Patients With Cancer With COVID-19: A Mathematical Dynamic Model for Latin America

**DOI:** 10.1200/GO.20.00156

**Published:** 2020-05-29

**Authors:** Alejandro Ruiz-Patiño, Oscar Arrieta, Luis E. Pino, Christian Rolfo, Luisa Ricaurte, Gonzalo Recondo, Zyanya-Lucia Zatarain-Barron, Luis Corrales, Claudio Martín, Feliciano Barrón, Carlos Vargas, Hernán Carranza, Jorge Otero, July Rodriguez, Carolina Sotelo, Lucia Viola, Alessandro Russo, Rafael Rosell, Andrés F. Cardona

**Affiliations:** ^1^ Foundation for Clinical and Applied Cancer Research, Bogotá, Colombia; ^2^ Molecular Oncology and Biology Systems Research Group, Universidad el Bosque, Bogotá, Colombia; ^3^ Thoracic Oncology Unit, Instituto Nacional de Cancerología, Mexico City, Mexico; ^4^ Oncology Department, Institute of Oncology, Fundación Santa Fe de Bogotá, Bogotá, Colombia; ^5^ Marlene and Stewart Comprehensive Cancer Center, Experimental Therapeutics Program, School of Medicine, University of Maryland, Baltimore, MD; ^6^ Center for Medical Education and Clinical Research, Buenos Aires, Argentina; ^7^ Department of Oncology, Centro de Investigación y Manejo del Cáncer, San José, Costa Rica; ^8^ Thoracic Oncology Unit, Alexander Fleming Institute, Buenos Aires, Argentina; ^9^ Clinical and Translational Oncology Group, Clínica del Country, Bogotá, Colombia; ^10^ Thoracic Oncology Unit, Fundación Neurmológica Colombiana, Bogotá, Colombia; ^11^ Medical Oncology Unit, Azienda Ospedaliera Papardo, Messina, Italy; ^12^ Coyote Research Group, Pangaea Oncology, Laboratory of Molecular Biology, Quiron-Dexeus University Institute, Barcelona, Spain; ^13^ Institut d’Investigació en Ciències Germans Trias i Pujol, and Institut Català d’Oncologia, Hospital Germans Trias i Pujol, Badalona, Spain

## Abstract

**PURPOSE:**

In the midst of a global pandemic, evidence suggests that similar to other severe respiratory viral infections, patients with cancer are at higher risk of becoming infected by COVID-19 and have a poorer prognosis.

**METHODS:**

We have modeled the mortality and the intensive care unit (ICU) requirement for the care of patients with cancer infected with COVID-19 in Latin America. A dynamic multistate Markov model was constructed. Transition probabilities were estimated on the basis of published reports for cumulative probability of complications. Basic reproductive number (R0) values were modeled with R using the EpiEstim package. Estimations of days of ICU requirement and absolute mortality were calculated by imputing number of cumulative cases in the Markov model.

**RESULTS:**

Estimated median time of ICU requirement was 12.7 days, median time to mortality was 16.3 days after infection, and median time to severe event was 8.1 days. Peak ICU occupancy for patients with cancer was calculated at 16 days after infection. Deterministic sensitivity analysis revealed an interval for mortality between 18.5% and 30.4%. With the actual incidence tendency, Latin America would be expected to lose approximately 111,725 patients with cancer to SARS-CoV-2 (range, 87,116-143,154 patients) by the 60th day since the start of the outbreak. Losses calculated vary between < 1% to 17.6% of all patients with cancer in the region.

**CONCLUSION:**

Cancer-related cases and deaths attributable to SARS-CoV-2 will put a great strain on health care systems in Latin America. Early implementation of interventions on the basis of data given by disease modeling could mitigate both infections and deaths among patients with cancer.

## INTRODUCTION

A new pathogen, identified as a novel coronavirus (SARS-CoV-2), triggered a pneumonia (COVID-19) outbreak in December 2019, starting in Wuhan, China, and spreading quickly to 31 provinces in China and > 186 countries worldwide. SARS-CoV-2 is a β-coronavirus. It shares a genetic sequence and viral structure with both severe acute respiratory syndrome coronavirus (70% similarity), which caused 349 deaths during 2002-2003 in China, and Middle East respiratory syndrome coronavirus (40% similarity).^1^ As of the March 23, 2020, a total of 81,507 confirmed cases of COVID-19, including 3,274 deaths, were reported in mainland China, Hong Kong, Macao, and Taiwan. A rapidly growing number of cases had also been reported worldwide. The situation was critical for health systems of Italy, Spain, and Iran, which together have had > 10,200 deaths. Mortality varies significantly according to country: 0.42% in Germany and 9.5% and 6.6% in Italy and Spain, respectively. Three and a half months after the start of the pandemic, 6,127 cases and 83 deaths have been diagnosed in Latin America, with a current mortality of 1.3%.^2^

**CONTEXT**

**Key Objective**
SARS-CoV-2 infection represents a substantial clinical issue in patients with cancer. However, the number of cases in Latin America has not reached a critical point, and the impact on deaths of oncologic patients has not been determined or estimated.
**Knowledge Generated**
Here, we demonstrate the impact on patients with cancer in terms of both intensive care unit requirements and projected deaths in a variety of scenarios, including a reduction, maintenance, and increase in the R0 value of COVID-19 in Latin America. These findings suggest that the reduction in the reproduction number is a feasible way to reduce cumulative mortality. In addition, with increased cancer disease burden in the region and with incidence and mortality expected to double by the year 2035, control of SARS-CoV-2 in the oncologic population should be a priority.
**Relevance**
These results should be used by decision makers and policymakers with the aim of mitigating the negative impact of the COVID-19 pandemic.

The number of COVID-19 cases is still rising. The median time from first symptoms to acute respiratory distress syndrome (ARDS) is 8 days (interquartile range, 6-12 days).^3^ The transition to ARDS occurs in many severe COVID-19 cases. A possible explanation for this rapid and serious deterioration is the cytokine release syndrome, or cytokine storm, an overproduction of immune cells and cytokines that leads to rapid multi-organ system failure and fatal damage to tissues of the lungs, kidney, and heart.^4^ Recently, Wu et al^5^ described that the main risk factors related to negative outcomes after COVID-19 infection are older age, neutrophilia, and organ and coagulation dysfunction. A recent integrative study that included information from 46,248 COVID-19 cases confirmed these findings, highlighting the risk of patients with cardiovascular comorbidity, hypertension, and chronic lung involvement.^6^

Similar to other severe respiratory viral infections, patients with cancer are more susceptible to COVID-19 infection because of their immunosuppression state caused by the malignancy and anticancer treatments.^7,8^ Liang et al^9^ reported 18 cases of patients with cancer and COVID-19 (1% of 1,590 patients); the most frequent neoplasm was lung cancer (28%). Four of 16 patients with cancer and COVID-19 had received chemotherapy or had undergone surgery in the past month, and the other 12 were survivors in routine follow-up after primary resection. Compared with patients without cancer, patients with cancer and COVID-19 were older (mean age, 63.1 years), were more likely to have a smoking history (22%), had more polypnea/dyspnea (47%), and had more severe baseline computed tomography scan manifestation (94%). Remarkably, patients with cancer were observed to have a higher risk of severe events compared with patients without cancer (39% *v* 8%) even after adjusting for other risk factors, including age, smoking history, and other comorbidities. This suggests that cancer history represents the highest risk factor for severe events. In addition, patients with cancer and COVID-19 deteriorated more rapidly (median time to severe events, 13 *v* 43 days; *P* < .0001).

Recently, Kucharski et al^10^ combined a stochastic transmission model with data on cases of COVID-19 in Wuhan and international cases that originated in Wuhan to estimate how transmission had varied over time since January 2020. In addition, Grasselli et al^11^ published a linear model forecast to estimate critical care utilization for the outbreak in Lombardy, Italy. In consideration of the importance of this information and the impact on public health for Latin America, we have modeled the mortality and intensive care unit (ICU) requirement for the care of Hispanic patients with cancer infected with COVID-19.

## METHODS

**FIG 1:**
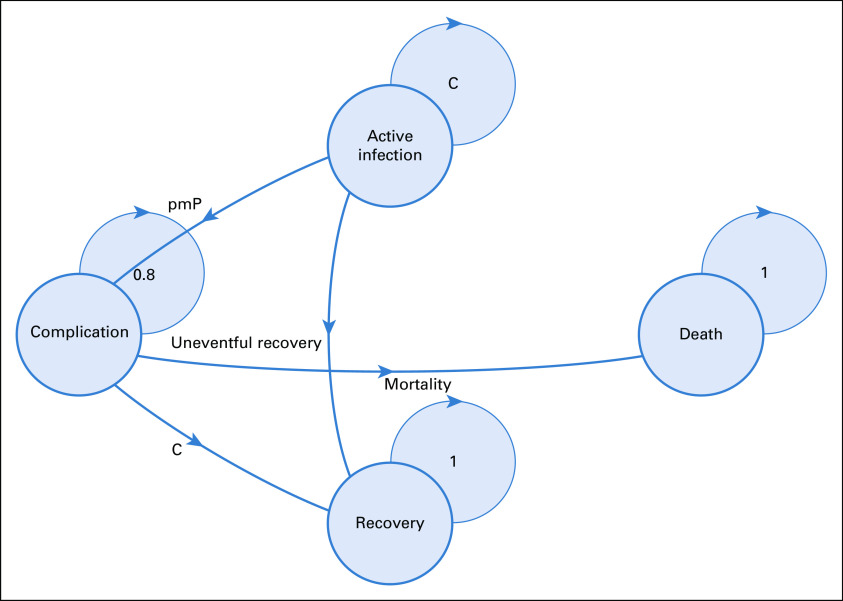
Disease state model. C, complementary probability; pmP, probability for complication.

To estimate the number of ICU requirements per day and per patient as well as expected mortality, a multistate Markov model was constructed. Disease modeling consisted of a simulated cohort of patients with cancer infected with SARS-CoV-2 that could potentially traverse 4 health states: actively infected without complications, complicated infections (ICU requirement), recovered from infection, and deceased. Mortality attributable to other aspects, such as disease progression, was not considered in the model because no report of this phenomenon was documented in the published cohorts.^12,13^ Figure 1 represents the model construction as well as the transition paths for patients. The model was constructed using the heemod package for R version 3.6.3 (The R Foundation, Vienna, Austria).

Transition probabilities were estimated on the basis of published reports for cumulative probability of complications, defined by requiring ICU, mechanical ventilation, or death. Because the behavior of probability of complication changes depending on the number of days after development of symptoms, a time-varying transition probability was fitted to a distribution obtained by the behavior of an infected cancer cohort.^8^ Probability of complication was estimated by extracting the number of patients at risk for each event and calculating a hazard function, which was later converted to describe transition probability. The difference between the observed and fitted distribution was estimated with the Kolmogorov-Smirnov test. The Data Supplement shows the estimated hazard function and fitted probability function. Mortality as well as uneventful recovery probabilities were estimated on the basis of published cohorts.^12,13^

The number of cancer cases among infected patients was estimated to be approximately 1.32% (95 CI, 0.98% to 1.66%).^8,12,13^ To estimate the full extent and number of possible cancer cases, hospitalizations in the ICU, and deaths, projections for all Latin American nations, where data were available, were constructed. Basic reproduction number (R0) values were modeled with R using the EpiEstim package. Data for parameter estimation was obtained from the Center for Systems Science and Engineering at Johns Hopkins University.^14,15^ The cutoff date was March 27, 2020. Serial interval distribution was modeled after a log-normal distribution with a mean value of 4.0 days (standard deviation, 2.9 days).^16^ The mean value of the last 5 days before the construction of this model was defined as R0 for each nation (R0n). After projecting 60 days into the future using a conservative R0 of 1.5, R0n, and 0.5 added to R0n, the total number of expected infected patients with cancer was calculated per day. Estimation of days of ICU requirement was calculated by imputing the number of cumulative cases in the Markov model. Absolute mortality was derived in the same manner.

### Data Sharing

All data, including implemented R code, are available upon request.

## RESULTS

**FIG 2:**
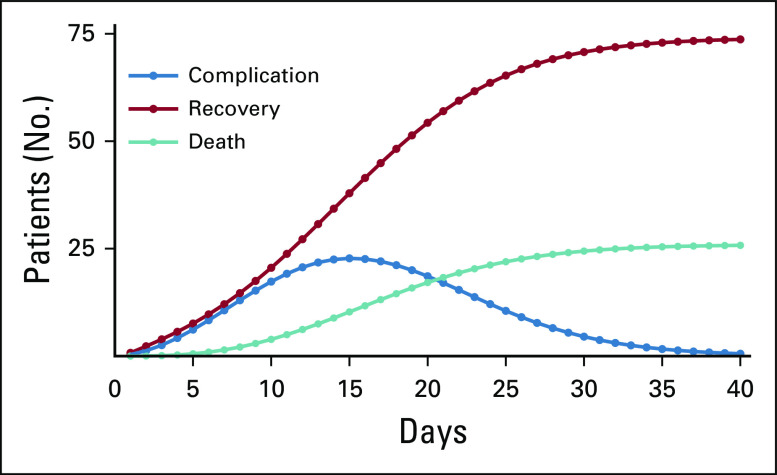
Cohort transition and dynamics among states.

By fitting a Weibull distribution (shape, 1.5; scale, 55; Akaike information criterion, 229.0469; *P* = .43) and protracting the remaining probabilities that aimed to reach an average mortality rate of 25%, behavior dynamics for patients with cancer infected with SARS-CoV-2 was estimated. Figure 2 shows a simulated cohort of patients and its transition between disease states.

As calculated by the model, compared with previously published metrics, median time from diagnosis to death (16.0 days; range, 9.0-22.3 days) and median time from diagnosis to severe event (7.0 days; range, 5.0-15.0 days)^13^ are concordant. R0 values were estimated for 15 nations for which data were available and enough time and cases since initial infection had occurred. Estimated incidence and R0 variation across days after the first case are represented for all included nations in the Data Supplement. Table 1 lists the included nations as well as the estimated R0 values and number of cases at day 60 of infection, depending on different R0 values. If a tendency of actual incidence continues, and therefore R0, Brazil would be the most affected nation with 27,089,291 infected patients in 60 days. Contrary to this, Uruguay would be the least affected with 1,387 cases. If strict social distancing and active isolation of infected individuals are enforced, with an expectation of a reduction of R0 to 1.5, Brazil would remain the most affected nation with 76,398 cases, but a reduction of 99.98% of total infections would be achieved. Bolivia would benefit the least from a reduction of R0, which would preventing 92.1% of total cases. A third scenario in which R0 increases is also presented, being catastrophic for all nations, especially Panama, resulting in the infection of the totality of its inhabitants. With regard to the oncologic population, the expected number of cases on the basis of the previously reported R0 are listed in Table 2. Case projection curves are presented for all included nations in the Data Supplement.

**TABLE 1:**
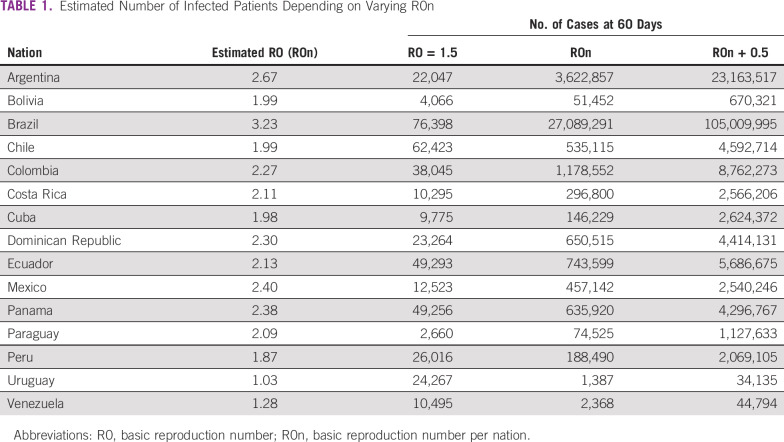
Estimated Number of Infected Patients Depending on Varying R0n

**TABLE 2:**
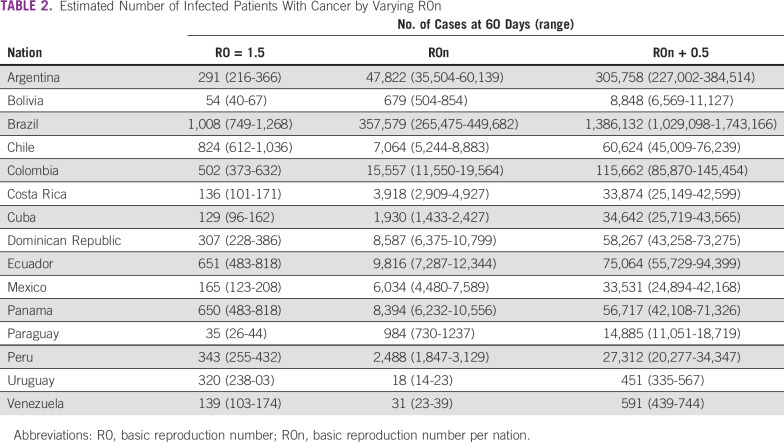
Estimated Number of Infected Patients With Cancer by Varying R0n

In addition, the expected number of ICU days per person computed on the basis of disease dynamics are listed in Appendix Table A1. With taking into account time to death, mortality, and then derived recovery rates and only considering ICU requirement and not a general hospitalization stay, the mean number of ICU days per person is approximately 4.1. Estimated mortality, by taking into account rate sensitivity analysis, is listed in Appendix Table A2. The number of deceased patients varies greatly across the region. Even in the highest projected R0, nations such as Uruguay and Venezuela are expected to have relatively few cases compared with other countries.

**FIG 3:**
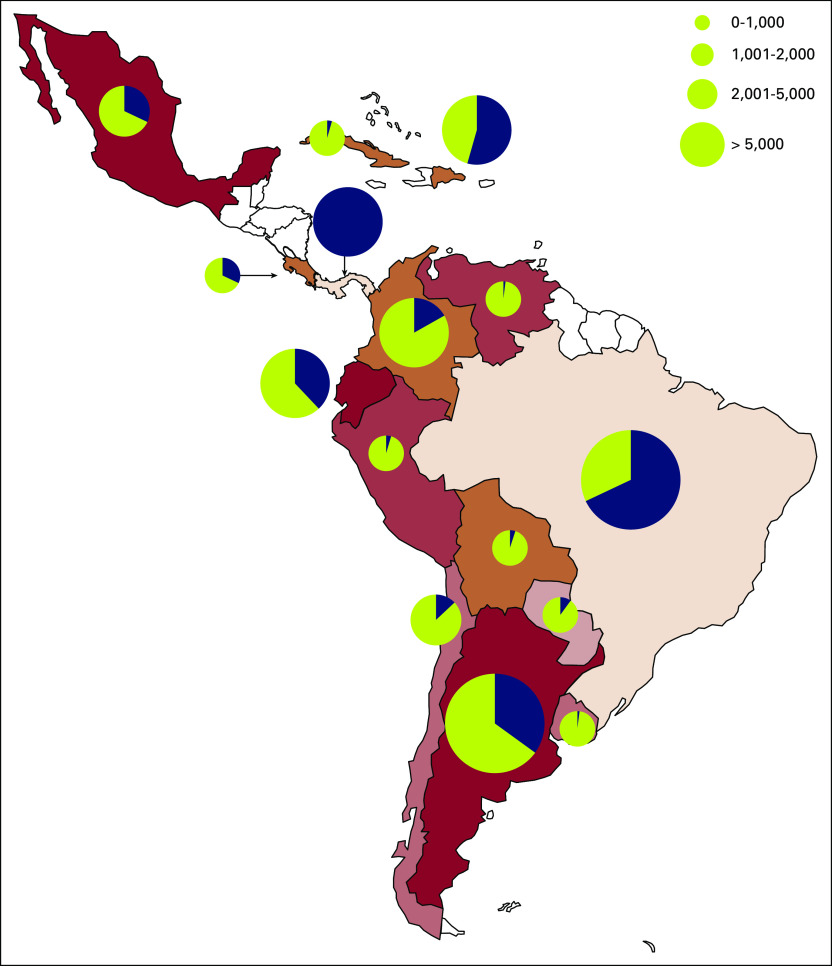
Map for Latin American distribution of cases among patients with cancer and expected mortality. COVID-19 mortality rate for patients with cancer estimated for Latin America. Circle size corresponds to number of cases. Percentage of blue for each nation corresponds to the proportion of all patients with cancer in each nation who could die as a result of SARS-CoV-2 on base 10. (A total blue circle therefore corresponds to 10% of all patients with cancer.)

By maintaining actual R0n values, Latin America as a region would be expected to lose approximately 111,725 patients with cancer to SARS-CoV-2 (range, 87,116-143,154 patients) by the 60th day since the start of the outbreak. With an estimated prevalence of cancer in the included nations of 3,133,806 individuals,^17^ the various scenarios put a loss of < 1% to 17.6% of all patients with cancer in the region (Fig 3).

## DISCUSSION

Cancer in Latin America represents an extensive disease burden. Projections estimate that by the year 2030, there will be an increase in cases of 72% with 78% mortality compared with 2012 data. By that date, it is expected for 1,831,300 new cancer cases and 1,061,500 cancer-related deaths to occur.^18^ With an estimate of 111,725 additional deaths of patients with cancer as a result of severe SARS-CoV-2 infection in the next 60 days, an increase of 18.5% in cancer-related deaths, compared with 2012 (603,300 deaths), is expected. These sobering predictions further highlight the need for a strict global registry of deceased patients with cancer in the setting of this pandemic to obtain reliable and accurate cancer statistics that will allow the assessment of health policies and their impact on cancer control and mortality.

Patients with cancer are indubitably more affected by SARS-CoV-2. In the nationwide analysis of patients with cancer infected in China conducted by Liang et al,^9^ an estimated prevalence of 1% of COVID-19 cases was identified. By comparing this value with the cancer incidence for 2015 in China, a relative over-representation of 300% was observed, which indicates a larger susceptibility to infection. A significant proportion of SARS-CoV-2 infections were suspected to have occurred in the health care setting.^3,7^ Patients with cancer are constantly exposed to health care environments as part of their routine care. This, in combination with an immunologic misbalance, could help to account for the larger incidence in the cancer population.

Another key aspect to be considered is the limited resources available to patients with cancer during these pandemic times. The expected reorganization of health care institutions to face the challenges of a massive influx of acute and complicated patients with COVID-19 will most likely have an impact on the health care delivered to nonrespiratory patients. For example, the availability and transport of stem cells for transplantation has faced several challenges. On the one hand, travel restrictions pose difficulty in the transportation of material and viability of the cells. On the other hand, donor consecution has been problematic because donors are required to assist in the screening process as well as spend time in hospitals during donation, increasing the risk of exposure to SARS-CoV-2.^19^ As more resources are allocated to treating critical patients, it is also understandable that shortages exist in other areas, although this has not been the case up to this moment.^19^ Anticancer treatments are associated with worse clinical outcomes when administered within 14 days of infection. Zhang et al^13^ showed a clear impact in severe event–free probability compared with patients in whom treatment was administered after that time frame (*P* = .037). Taking this into consideration, the decision to withhold treatments could be an observable phenomenon, especially in settings where the risk-benefit of administration is not extensive, such as adjuvant treatments in low-risk tumors. Fortunately, recommendations such as home delivery of treatments, modification of treatment schedules, and specialty center isolation could be successfully implemented.^20^

The utility of modeling disease is extensive and has been widely documented. Its main goal is to predict and establish disease behavior. The results from these estimations gain in importance in times of pandemics because they offer fundamental data useful for decision making and implementation of health policies meant to control, mitigate, and prevent disease outbreaks. There are several challenges to disease modeling in real time. First, there are ethical issues related to the use of surveillance data that can generate data-sharing limitations. Opportunely, data availability on COVID-19 for modeling and monitoring has not been an issue. On the other hand, model interpretation is also a complex process in which misinterpretation can lead to erroneous decisions.^21^ Another delicate aspect to consider is data quality and adequate reports. Although data are available for all examined nations, it is difficult to evaluate the degree of under-registration samples. Testing strategy implementation is different in each nation, and accessibility is not universal.^22^ Each Latin American nation has conducted different plans of action for the detection and definition of candidates for the application of tests. Taking this into consideration, the number of incident cases could be unreliable, and therefore an absolute estimation is uncertain. To compensate for this aspect, this study offers intervals for which decision makers could influence their conduct. Other difficulties, especially in the estimation of mortality, lie on the different demographics of cancer cohorts. Patients with cancer tend to be older and to suffer from more comorbidities than the average COVID-19 patient, with an increased risk of complications and death beyond the cancer comorbidity itself.^9,13^ Estimation of the effect of variability of cohort composition is difficult with the limited information available of infected patients with cancer. Furthermore, Latin America has a different demographic of patients with cancer, especially with an infection-related diagnosis, which leads to a younger oncologic population.^18^ To compensate for this variation, deterministic sensitivity analysis in mortality was incorporated, and values within ranges were given. Although ideally, other forms of sensitivity analyses can be conducted, because of variable uncertainty, these approximations could be equivocal, especially in resampling distributions among demographic characteristics.

Interesting discrepancies can also be observed. In the case of Mexico, a nation with an estimated R0 of 2.4, a relatively smaller number of expected cases is observed. Taking into account a low initial R0 value as well as a small number of cases at day 28, after the projections were estimated, only 32 days of exponential growth at that rate were observed. Compared with Ecuador, in which 1,403 cases were diagnosed at day 26, results in 34 days of growth with an R0 of 2.13 yielded a much higher case number at day 60. Possible explanations for an initial low count of cases could include underdiagnosis of early cases.

Other aspects to consider include ventilator and ICU bed availability. To this day, and to our knowledge, no rigorous and recent publication discussing the situation on ICU availability for Latin America exists. Conducts derived from the information contained in this article should fall to local health authorities. With regard to interventions that could mitigate the impact of this pandemic, social distancing and patient isolation seem to be the most effective ways of diminishing R0 values and reducing the impact on the number of infections, which protracts this benefit toward patients with cancer.^10^

In conclusion, cancer-related cases and deaths attributable to SARS-CoV-2 will put a great strain on health care systems in Latin America. Although up to this point experiences from around the world have shown that the limits of health care delivery will be pushed, patients with cancer are at a greater risk for complications. On a more positive note, the early application of interventions on the basis of data given by disease modeling could mitigate both infections and deaths among patients with cancer.

## Figures and Tables

**TABLE A1:**
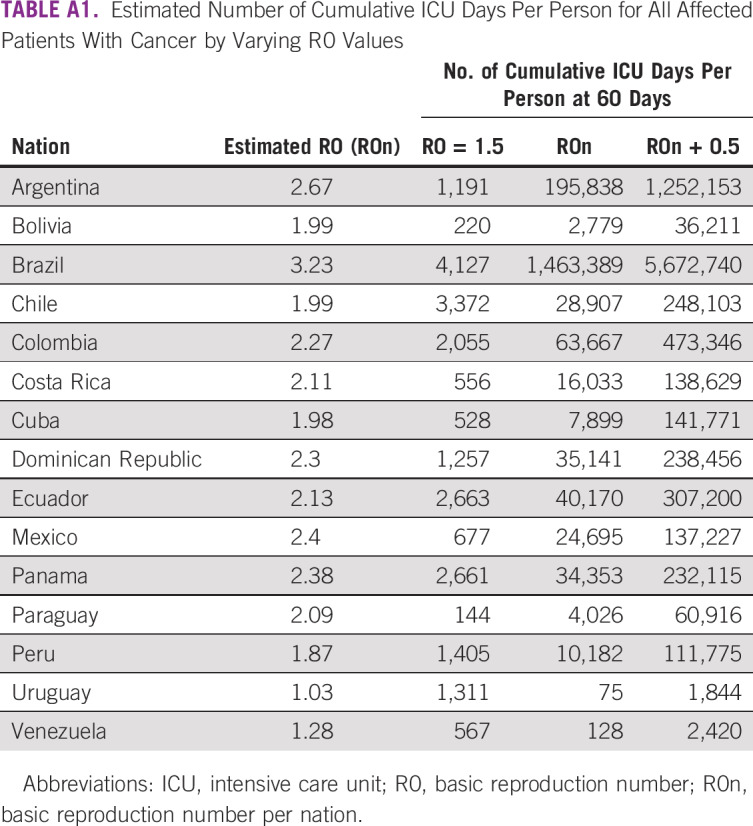
Estimated Number of Cumulative ICU Days Per Person for All Affected Patients With Cancer by Varying R0 Values

**TABLE A2:**
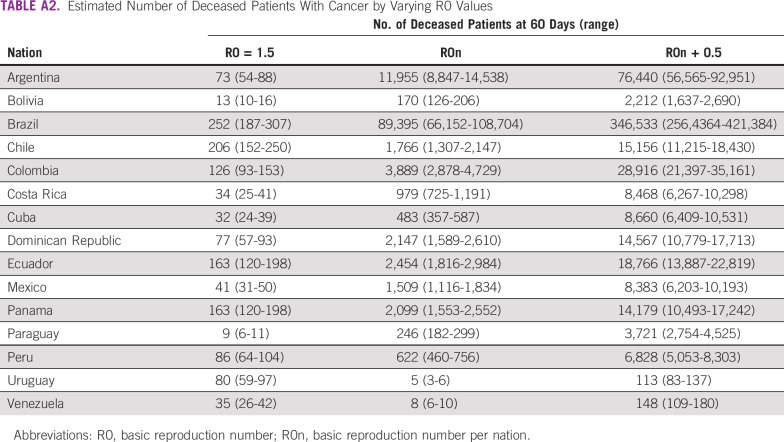
Estimated Number of Deceased Patients With Cancer by Varying R0 Values
